# Fat King Penguins Are Less Steady on Their Feet

**DOI:** 10.1371/journal.pone.0147784

**Published:** 2016-02-17

**Authors:** Astrid S. T. Willener, Yves Handrich, Lewis G. Halsey, Siobhán Strike

**Affiliations:** 1 Department of Life Sciences, University of Roehampton, London, United Kingdom; 2 Université de Strasbourg, Institut Pluridisciplinaire Hubert CURIEN, Strasbourg, France; 3 Centre National de la Recherche Scientifique, Unité Mixte de Recherche 7178, Strasbourg, France; National Cancer Institute, UNITED STATES

## Abstract

Returning to the shore after a feeding sojourn at sea, king penguins often undertake a relatively long terrestrial journey to the breeding colony carrying a heavy, mostly frontal, accumulation of fat along with food in the stomach for chick-provisioning. There they must survive a fasting period of up to a month in duration, during which their complete reliance on endogenous energy stores results in a dramatic loss in body mass. Our aim was to determine if the king penguin’s walking gait changes with variations in body mass. We investigated this by walking king penguins on a treadmill while instrumented with an acceleration data logger. The stride frequency, dynamic body acceleration (DBA) and posture of fat (pre-fasting; 13.2 kg) and slim (post fasting; 11 kg) king penguins were assessed while they walked at the same speed (1.4km/h) on a treadmill. Paired statistical tests indicated no evidence for a difference in dynamic body acceleration or stride frequency between the two body masses however there was substantially less variability in both leaning angle and the leaning amplitude of the body when the birds were slimmer. Furthermore, there was some evidence that the slimmer birds exhibited a decrease in waddling amplitude. We suggest the increase in variability of both leaning angle and amplitude, as well as a possibly greater variability in the waddling amplitude, is likely to result from the frontal fat accumulation when the birds are heavier, which may move the centre of mass anteriorly, resulting in a less stable upright posture. This study is the first to use accelerometry to better understand the gait of a species within a specific ecological context: the considerable body mass change exhibited by king penguins.

## Introduction

The walking biomechanics of a penguin are of interest both because they represent a waddling gait and because penguin locomotion adaptations seem focussed more on swimming than walking [1–4]. Pinshow et al. (1976a) recorded energetics and biomechanical data for three penguin species walking on a treadmill at various speeds. They reported that penguin pedestrian locomotion is energetically expensive relative to other species with similar body masses and suggested that this high energy cost was explained by inefficiencies of the waddling gait [2]. However, subsequent research has suggested that penguin waddling is in fact an effective mechanism for energy transfer between steps and that for them walking is expensive because of their short legs [5].

The short effective leg length of penguins may have evolved to enhance swimming capability and a possible reduction of heat loss, potentially at the expense of walking efficacy with the former mode of locomotion arguably being more intimately related with survival and reproductive success. When swimming, their short legs, placed in line with their body, ensure they have a compact, hydrodynamic and well insulated body. Possibly, such legs demand considerable lateral movement of the trunk to facilitate walking, equivalent to a Trendelenburg gait in humans [6]. However, in humans this side to side movement is often reflected in a wide step, and this step width variability has been associated with older people who are at risk of falling [7–9]. In contrast to humans, king penguins exhibit reduced variability in the frontal plane evidenced by a small standard deviation in step width compared to step length [10], which may be related to stability. However, beyond this, only limited research has been conducted on the biomechanics of penguin walking.

An aspect of their waddling gait that has not been explored is how it might vary with body mass. After returning ashore from a foraging trip at sea, some king penguins need to walk a relatively long distance to reach their breeding colony, and while doing so they are carrying a heavy, mostly frontal, accumulation of fat and ingested prey for their chicks; essential energy stores for their long fast on land. During this period of fasting, which can last up to a month, king penguins lose about one quarter of their body mass as fat (predominantly around the brood patch) and muscle (predominantly the pectorals) [3, 4, 11–13]. Obese humans choose to walk at slower self-selected speeds with shorter steps and longer stance durations than do people of normal body mass [14], while pregnant women adjust their gait by increasing step width and decreasing step length [15]. As far as we are aware, however, there are as yet no investigations into whether and how gait kinematics vary with body mass in other species.

To better understand the biomechanics of pedestrian locomotion in penguins and how these might change with their naturally high variability in body mass, the accelerometry method [16] was applied to king penguins walking on a treadmill in both pre-fasting and post fasting states, which are associated with considerable differences in body mass. The aims of this study were to determine if there were differences in the birds when they were heavy compared to when they were light in terms of (1) the tri-dimensional acceleration of their trunk (2) their stride frequency and (3) their posture angles.

## Materials and Methods

### Birds

Ten king penguins in courtship identified as males from their behaviour [11] and selected for high body masses (>12 kg), were captured near the shoreline at the edge of the colony. They were kept for 14 days in a 2m^2^ pen while they fasted, thus enabling a paired experimental design whereby data were collected from the same individual at two body masses (day 0: body mass mean = 13.2 ± 0.6 kg; day 14: 11.0± 0.5 kg). They were tested for their ability to walk on a treadmill and trained to do so during at least two sessions of walking, each for approximately 10 minutes, before data collection commenced.

### Fieldwork and ethics

The study was undertaken during one austral summer season (2010–2011) within the king penguin colony at “Baie du Marin” on Possession Island, Crozet Archipelago (46°25’S; 51°52’E). All procedures used in the present study were approved by the “comité d’éthique pour l’expérimentation animale Midi-Pyrénées, supported by the IPEV Réf MP/11/20/04/10. Authorisations for the experiments were delivered by the Comité de l’Environnement Polaire”, Terres Australes et Antarctiques Françaises (permit n°2010–71 of the 3^rd^ of September 2010). The requirements of the United Kingdom (Scientific Procedures) Act 1986 were followed.

### Tri-axial accelerometer

A tri-axial accelerometer (Macrologger FCM 85x35x18 mm, 80 g. (conceived and built by P. Medina and R. Laesser, Bio-logging development team, CNRS-IPHC, Strasbourg, France) was firmly attached to the feathers of each penguin with tape (Tesa^®^ 4651) on the bird’s back, in line with the spinal column, at the height of the hip, and recorded at 32.5Hz. The junction between the pelvis and the spine (S1-L5 in humans) was chosen as the height of attachment. This is a good location to ensure consistent placement. This location is in front of the synsacrum, an avian anatomical structure where the spinal column is strongly attached to the sacrum, and is also a relatively fat-free bony surface, serving to minimise signal noise in the quantification of trunk motion.

Before data collection, the bird was required to walk for 5 minutes to habituate, which was followed by a resting period of 5 minutes. Then, accelerometer data were collected for 10 minutes while the bird was walking at a speed of 1.4 km/h (the modal speed within the breeding colony; pers. ob.).

### Data processing

#### Static body acceleration and postural angles

Each of the three accelerometers in a tri-axial logger attached to an animal is subjected to two kinds of events: (a) changes in the relative orientation of its axis to that of gravity (which is always vertical) due to postural changes of the animal, (b) dynamic movement due to dynamic movement of the animal. These two events result in changes in the value of the acceleration detected by the accelerometer, but their effects can be separated because postural changes typically occur at a lower frequency than do changes associated with dynamic movement. Gravity is recorded from 0 g when a stationary accelerometer is horizontal to the ground, to 1 g when vertical. This component, referred to as the Static Body Acceleration (SBA), allows the animals’ posture to be calculated [17].

**Fig 1:**
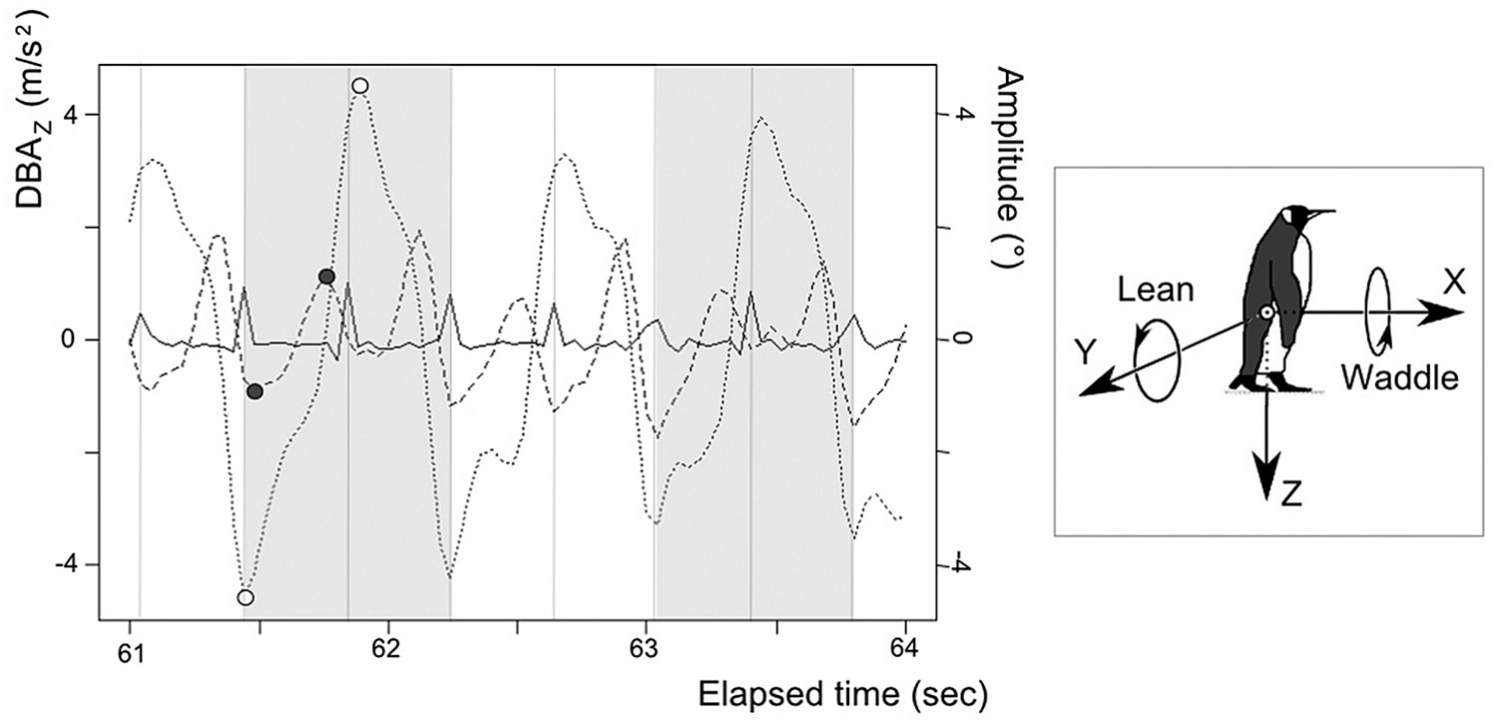
Dynamic body acceleration recorded over time in a king penguin in the z (vertical) axis (full line), along with waddling angle amplitude (pointed line; positive numbers mean right incline, negative number left incline) and backward leaning angle amplitude (dashed line). A grey shaded area followed by an unshaded area comprises a stride of the left leg, starting at the initial contact of the foot with the ground, represented by a grey vertical line. The maximum and minimum leaning angles within two initial contacts (i.e. two ‘steps’) are represented by black circles, while the maximum and the minimum waddling angle during one stride are represented by white circles. (Modified from [16])

For each axis, this static component was mathematically extracted from the corresponding raw acceleration data (rawA_x_, rawA_y_ and rawA_z_) by a first-order, low-pass Butterworth filter, at 1.25Hz, where the Vectorial norm of the Static Body Acceleration ($VeSBA\;=\;\sqrt[{2}]{{{SBA}_{x}}^{2}+{{SBA}_{y}}^{2}+\;{{SBA}_{z}}^{2}}$), equals 1 g [18] when the accelerometer is not subject to dynamic movement. In the second step, it is possible to calculate the two postural angles, defined by leaning ('pitch') and waddling ('roll') angles via trigonometry using the two opposite axes. Leaning angle has been calculated using data from axis X and VeSBA, and waddling angle was calculated from axes Y and Z [see details in [16] and Fig 1].

#### Dynamic Body Acceleration (DBA) and overall body motion

**Fig 2:**
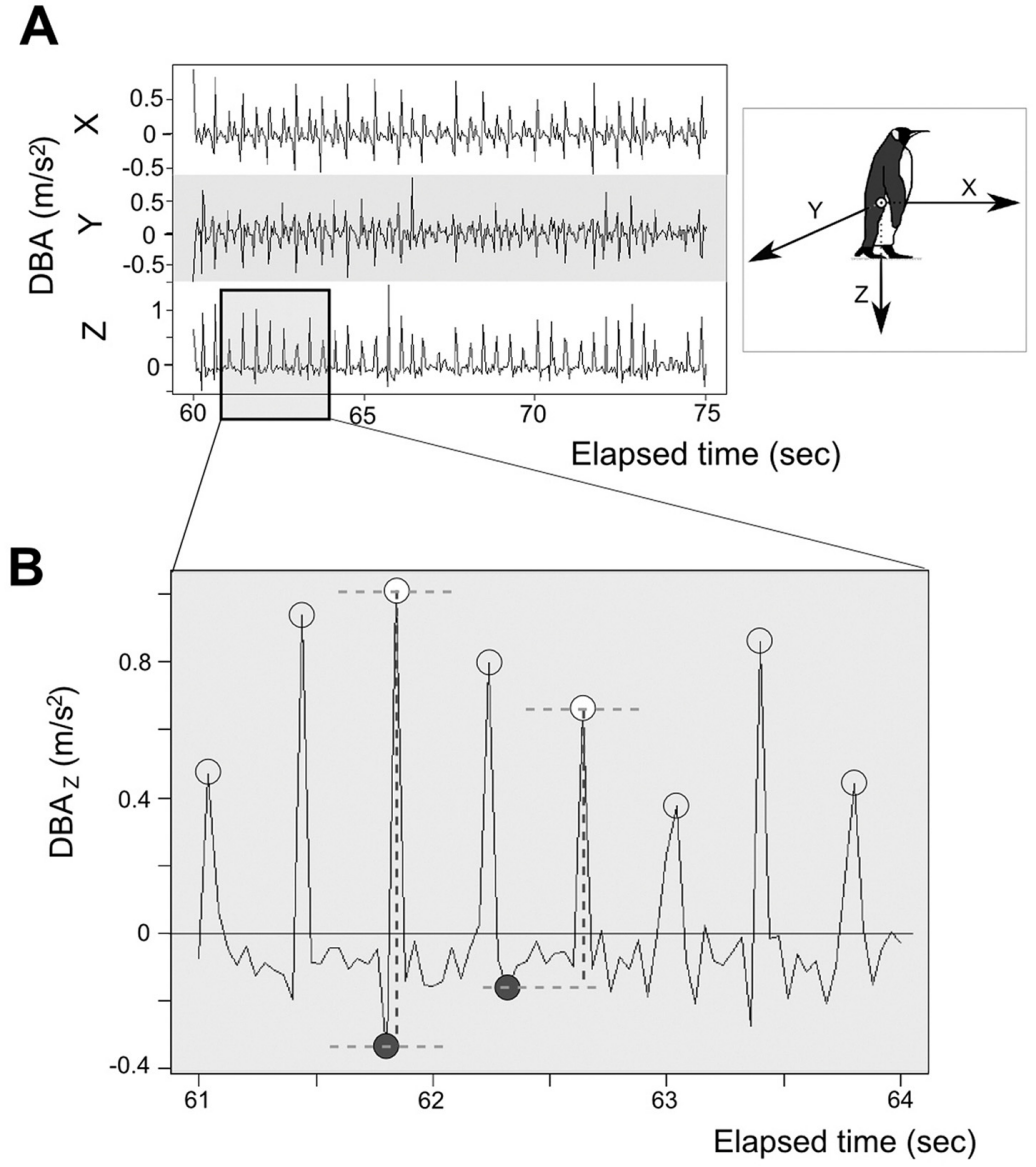
A. Example of dynamic body acceleration (DBA) data in three axes for a king penguin walking during 15 seconds. B. The DBA data for the z axis over three seconds of the same data set. Open circles represent maximum values and closed circles indicate minimum values identified by an automated, custom-written program. These maxima represent the initial contact of one of the king penguin’s feet with the ground. The dashed lines indicate the calculations of DBA amplitude based on these maximum and minimum values. **Right:** Visual representation of the DBA axes and angle movements (Modified from [16])

Acceleration due to the change of velocity of the animal to which the logger is attached can be referred to as Dynamic Body Acceleration (DBA_x_, DBA_y_, DBA_z_,[17]). This part of the signal corresponds mathematically on each axis to the difference between raw and Static Body Acceleration (e.g. DBA_x_ = RawA_x_- SBA_x_) [See details in [16] and Fig 2].

### Overall gait: DBA amplitude

Traces of DBA manifest as a sequence of peaks, which can be described by a maximum value followed by a minimum value; these were calculated with an algorithm within a custom-written script for MATLAB (MATLAB, 2010) (Fig 2). The amplitude was calculated as the difference between the minimum and the subsequent maximum. Finally the means and variability (SD) of the different DBA amplitudes were calculated for each individual, using R [19]. The first minute of the 10-minute walking session was removed in all cases as some birds presented an irregular walk at the start of the experiment. N = 7 for all the analyses due to three data loggers failing to record.

### Strides

The maximal DBA values on the Z-axis (i.e. DBA_z_; Fig 1) represent the highest instances of vertical acceleration, which are associated with the initial contact of a foot with the ground (Fig 2). Thus each maximum represents a step made by the instrumented bird. As one stride is composed of two steps, the stride frequency is half the calculated step frequency.

### Posture: waddling and leaning

The posture (leaning and waddling) of the penguins while walking was determined [see details in [16] and Fig 1]. To quantify degree of leaning, two parameters were defined: the amplitude of peak forwards and backwards leaning (i.e. max-min) and the mean leaning angle (i.e. the mean min to max). To quantify waddling, a single parameter was calculated: the amplitude of peak left and right leaning. Vertical DBA was used to define each stride ensuring that only one minimum and one maximum angle of waddling in each stride was calculated and to determine the minimum and maximum angles of leaning in each step (Fig 1). For the DBA amplitude, the mean and variability (i.e. SD) for each of these postural parameters were calculated per individual.

### Statistical analysis

Pairwise Wilcoxon signed-rank tests were undertaken to test for pair-wise differences in median values for each parameter between the birds when heavy and when light, using R [19]. The P value associated with these tests is interpreted as a continuous variable indicating the strength of evidence against the null hypothesis [20–22].

## Results

### Global change of gait -DBA

There was no evidence for an effect of body mass on the mean amplitude of any DBA axis (p = 0.94, 0.30 and 0.94, for DBA_x_, DBA_y_, DBA_z_ respectively) or for variability (i.e. standard deviation) in their amplitude (p = 0.30, 0.58 and 0.30, for DBA_x_, DBA_y_, DBA_z_ respectively) (Table 1).

**Table 1:** The mean amplitude of DBA in each axis measured by an accelerometry instrumented to king penguins while walking at 1.4 km/hour, when light and when heavy.

	Axis		Body Mass		P value
			~13.2 kg	~11.0 kg	
**Amplitude [*g*]**	**DBA**_**X**_	Mean ± SD	0.38 ± 0.05	0.40 ± 0.08	0.94
		Variability ± SD	0.17 ± 0.04	0.14 ± 0.06	0.30
	**DBA**_**Y**_	Mean ± SD	0.58 ± 0.06	0.52 ± 0.08	0.30
		Variability ± SD	0.24 ± 0.05	0.22 ± 0.05	0.58
	**DBA**_**Z**_	Mean ± SD	0.69 ± 0.16	0.66 ± 0.16	0.94
		Variability ± SD	0.23 ± 0.06	0.20 ± 0.07	0.30

### Stride parameters

There was no evidence for a difference in stride frequency between the high and low body mass conditions (stride frequency ± SD = 1.27± 0.11[stride.s^-1^] and 1.26± 0.09 [stride.s^-1^], respectively, p = 0.81).

### Posture: Waddling and leaning

There was no evidence for a difference in mean waddling amplitude (p = 0.30), leaning amplitude (p = 1.00), or leaning angle (p = 0.81) between body mass conditions (Table 2). The variability of both the leaning angle and leaning amplitude were lower in the lighter birds (p = 0.03 and 0.03, respectively), while there was some evidence that variability in waddling amplitude was also lower (p = 0.08).

**Table 2:** The mean and the variability of waddling and leaning amplitudes, and the mean leaning angle, of king penguins while walking at 1.4 km/hour, when light and when heavy.

Waddling parameters	Body Mass		P value
	~13.2 kg	~11.0 kg	
**Mean waddling amplitude [°]**			
Mean ± SD	8.51 ± 1.68	7.38 ± 2.33	0.30
Variability ± SD	2.24 ± 1.34	1.45 ± 0.70	0.08
**Mean leaning amplitude [°]**			
Mean ± SD	1.90 ± 0.49	1.85 ± 0.61	1
Variability ± SD	1.02 ± 0.44	0.72 ± 0.38	0.03
**Mean leaning angle [°]**			
Mean ± SD	0.04 ± 0.05	0.03 ± 0.08	0.81
Variability ± SD	0.40 ± 0.15	0.30 ± 0.15	0.03

## Discussion

At the imposed walking speed, king penguins do not exhibit major differences in their walking biomechanics whether heavy or light. Stride frequency did not change and nor did any of the three dynamic body accelerations recorded by the accelerometer attached to the birds. Furthermore, waddling amplitude, leaning amplitude and leaning angle all remained fairly constant across the two body masses. However, some differences were uncovered; in particular there was good evidence that variability in the leaning angle and leaning amplitude, and some evidence that the waddling amplitude, were lower when the birds were lighter. These results indicate that heavier king penguins have a higher frontal and sagittal instability; they are less stable walkers than when they are lighter.

**Fig 3:**
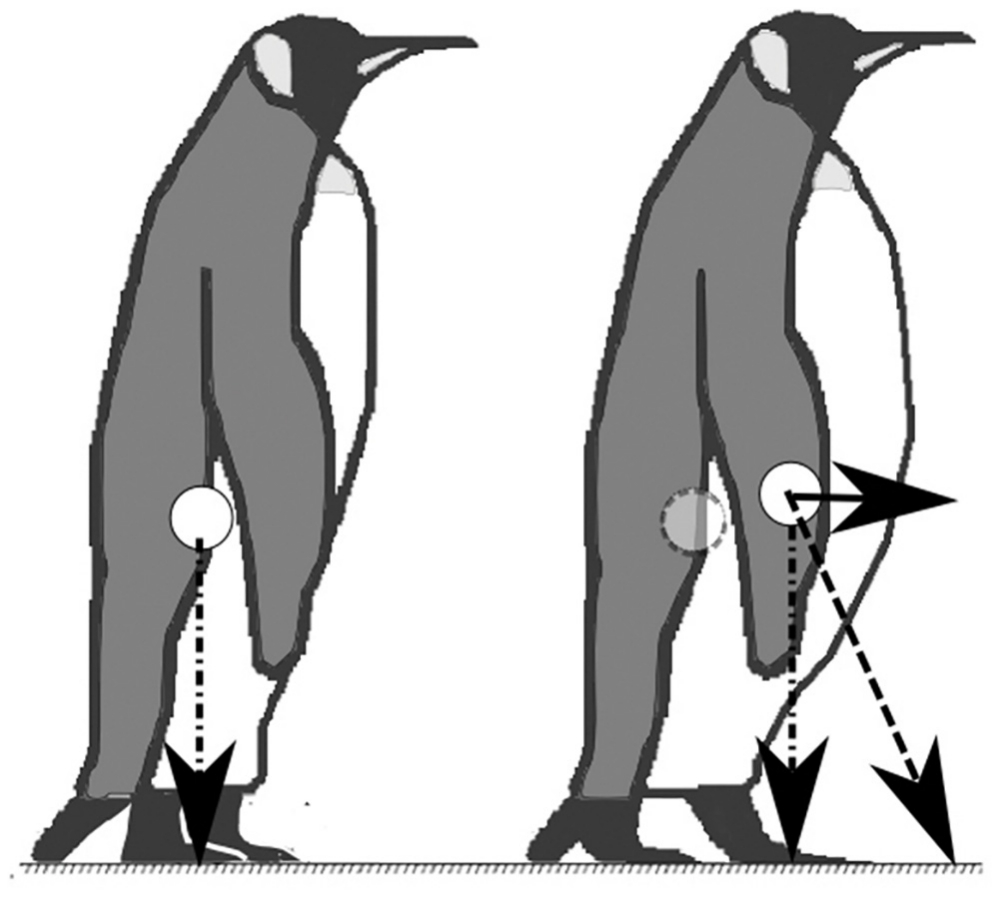
Theoretical change in the location of the centre of mass between light (left) and heavy (right) king penguins in a standing posture. This difference will produce a resultant force in heavy birds which may cause it to fall forward during each step. The greater momentum associated with this increased mass may cause an increase in variability in trunk motion.

King penguins that have returned from sea must have gained substantial endogenous energy stores to sustain the subsequent period of fasting onshore. The start of fasting in emperor penguins coincides with an abrupt reduction in abdominal fat, while subcutaneous fat accumulation decreases more slowly, and throughout the fasting period [4], and this is likely to be similar in the congeneric king penguin. This accumulation of anterior fat may shift the position of the bird’s centre of mass forwards in the sagittal plane (Fig 3), although if so we did not find evidence that this affected leaning angle during walking. Nonetheless, such fat accumulation may explain the possible increase in both leaning angle variability and amplitude variability of walking king penguins when they are heavier, as it is more difficult to control this larger mass and its associated momentum.

This is supported by the observation that heavy king penguins returning from the sea are more likely to fall than are lighter ones that have been fasting (Pers. Obs.). However, in turn it is perhaps surprising that the mean leaning angle of the birds does not differ with their body mass. Further research is needed to assess the effect of the location of the centre of mass relative to the feet/flippers and the effect that this distance may have on the linear and angular momentum of the trunk.

As mentioned, our results regarding stride frequency show that, contrary to obese and pregnant humans [14, 15], king penguins do not change their stride frequency with a change of body mass. Although the waddling gait may appear otherwise, it has in fact been demonstrated to impart stability. Kurz et al. (2008) found evidence of greater consistency in the stride width than the stride length of walking penguins, which may be explained by the waddling gait imparting lateral control [10]. However, the present results do not indicate that king penguins then adapt their waddle in concert with changes in their mass to optimise stability at different body weights. Obese humans alter hip, knee and ankle motion to facilitate walking [23–25], however for king penguins, due to their relatively short limbs and long flippers and trunks, their capacity to optimise the movements is presumably limited. We therefore hypothesised that heavier king penguins must produce a greater impulse to maintain the given walking speed by increasing the degree of waddle in their gait (Trendelenburg-type). Interestingly, our results did not support this hypothesis as our penguins maintained a similar waddling amplitude in both body mass conditions. Potentially, the penguins adapt their gait by increasing the rotation about the vertical axis, which may enable them to sustain their step length, however we cannot explore this line of enquiry without a gyroscope to quantify, the amplitude of the body’s yaw (rotation around the vertical z-axis). Furthermore, in the present study walking speed was not volitional but rather imposed; birds may adapt different speeds depending on their mass. However the treadmill speed chosen was that at which the penguins at both masses walked most fluently and is similar to the modal walking speeds observed within the colony [3]. Furthermore, the birds were chosen and trained to get used to walk fluently on the treadmill at this speed.

To summarise, the present research used accelerometry to quantify the biomechanics of the penguin walking gait. The results demonstrate that the mean acceleration of the trunk when a king penguin is walking at a fixed velocity does not vary with body mass. However, the variability in trunk acceleration tends to be higher in heavier king penguins. Further research, such as investigating different walking speeds and including gyroscope recordings, may elucidate biomechanical adaptations developed by this primarily aquatic bird to its terrestrial environment, which can involve carrying extensive body fat reserves over distance. More broadly, accelerometry shows great potential for gaining quantified insights into the gait biomechanics of animals.
